# Phylogenetic relationship and characterization of the complete mitochondrial genome of *Mylabris calida* (Coleoptera:Meloidae)

**DOI:** 10.1080/23802359.2020.1823276

**Published:** 2020-10-05

**Authors:** Ming Jiang, Qian Wei, Wenqiang Wang

**Affiliations:** a College of Life Sciences, Yan’an University, Shaanxi, Yan’an, China; b Shaanxi Engineering and Technological Research Center for Conservation and Utilization of Regional Biological Resources, Yan’an University, Shaanxi, Yan’an, China; c Research Center for Resource Polypeptide Drugs & College of Life Sciences, Yan’an University, Shaanxi, China

**Keywords:** Meloidae, mitochondrial genome, *Mylabris calida*, phylogenetic analysis

## Abstract

Beetle genus *Mylabris* (Meloidae) was described by Fabricius (1775) and had been well known due to its relevance to traditional medicine (e.g., cantharidin production). Here, we sequenced and annotated the mitochondrial genome (mitogenome) of *Mylabris calida*, one of species within *Mylabris*. This mitogenome was 15,149 bp long and encoded 13 protein-coding genes (PCGs), 22 transfer RNA genes (tRNAs) and two ribosomal RNA unit genes (rRNAs). All 13 PCGs were initiated by the ATN (ATG, ATT and ATA) codon. All PCGs terminated with the stop codon TAA or TAG except for *cox1*, *cox2*, *nad5* and *nad4* which end with the incomplete codon T—. Phylogenetic analysis showed that *M. calida* got together with the same genus species *Mylabris* sp., *M. aulica* and three *Hycleus* species (*H. cichorii*, *H. phaleratus* and *H. marcipoli*), indicating *Mylabris* has a closer relationship with *Hycleus* than other genera within Meloidae.

Meloidae, commonly known as blister beetles, is relatively well known in the production of cantharidin, parasitoid biology of its larval phases and biological literature due to its hypermetabolic larval development (Bologna et al. 2008). Mylabrini is the most speciose tribe of Meloidae, with approximately 750 described species being assigned to 11 genera (Pan and Bologna 2014).

Specimens of *M. calida* were collected from Yan’an City, Shaanxi Province, China (36°34′N, 109°41′E, May 2019) and were stored in Entomological Museum of Yan’an University (Accession number YAU-E-MC08). After morphological identification, total genomic DNA was extracted from tissues using DNeasy DNA Extraction kit (Qiagen). The mitogenome sequence of *M. calida* was generated using Illumina HiSeq 2500 Sequencing System. In total, 6.4 G raw reads were obtained, quality-trimmed, and assembled using MITObim v 1.7 (Hahn et al. 2013). By comparison with the homologous sequences of other Meloidae species from GenBank, the mitogenome of *M. calida* was annotated using software GENEIOUS R8 (Biomatters Ltd., Auckland, New Zealand).

The nearly complete mitogenome of *M. calida* is 15,149 bp (Genbank accession, MT880604). It contains 13 protein-coding genes (PCGs), 22 tRNA genes, two rRNA genes, and one partial non-coding AT-rich region. Gene order was conserved and identical to that of *Drosophila yakuba* and to most other previously sequenced Meloidae species (Du et al. 2016, 2017; Jie et al. 2016; Han et al. 2020). The overall base composition of the mitogenome was estimated to be A 37.5%, T 34.8%, C 16.9% and G 10.8%, with a high A + T content of 72.3%. All 13 PCGs of *M. calida* have the conventional ATN start codons for invertebrate mitochondrial PCGs (seven ATT, five ATG and one ATA). Most of the PCGs terminate with the stop codon TAA or TAG, whereas *cox1*, *cox2*, *nad5* and *nad4* end with the incomplete codon T. The 22 tRNA genes vary from 59 bp (*trnS1*) to 71 bp (*trnK*). Two rRNA genes (*rrnL* and *rrnS*) locate at *trnL1*/*trnV* and *trnV*/control region, respectively. The lengths of *rrnL* and *rrnS* in *M. calida* are 1,278 and 767 bp, with the AT contents of 76.1% and 74.1%, respectively.

Phylogenetic analysis was performed based on the nucleotide sequences of 13 PCGs from 16 Coleoptera species. Phylogenetic tree was constructed through raxmlGUI 1.5 (Silvestro and Michalak 2012). Results showed that the new sequenced species *M. calida* got together with the same genus species *Mylabris* sp., *M. aulica* and three *Hycleus* species (*H. cichorii*, *H. phaleratus* and *H. marcipoli*), indicating *Mylabris* has a closer relationship with *Hycleus* than other genera within Meloidae (Figure 1). In conclusion, the mitogenome of *M. calida* is sequenced in this study and can provide essential DNA molecular data for further phylogenetic and evolutionary analysis of Meloidae.

**Figure 1.**
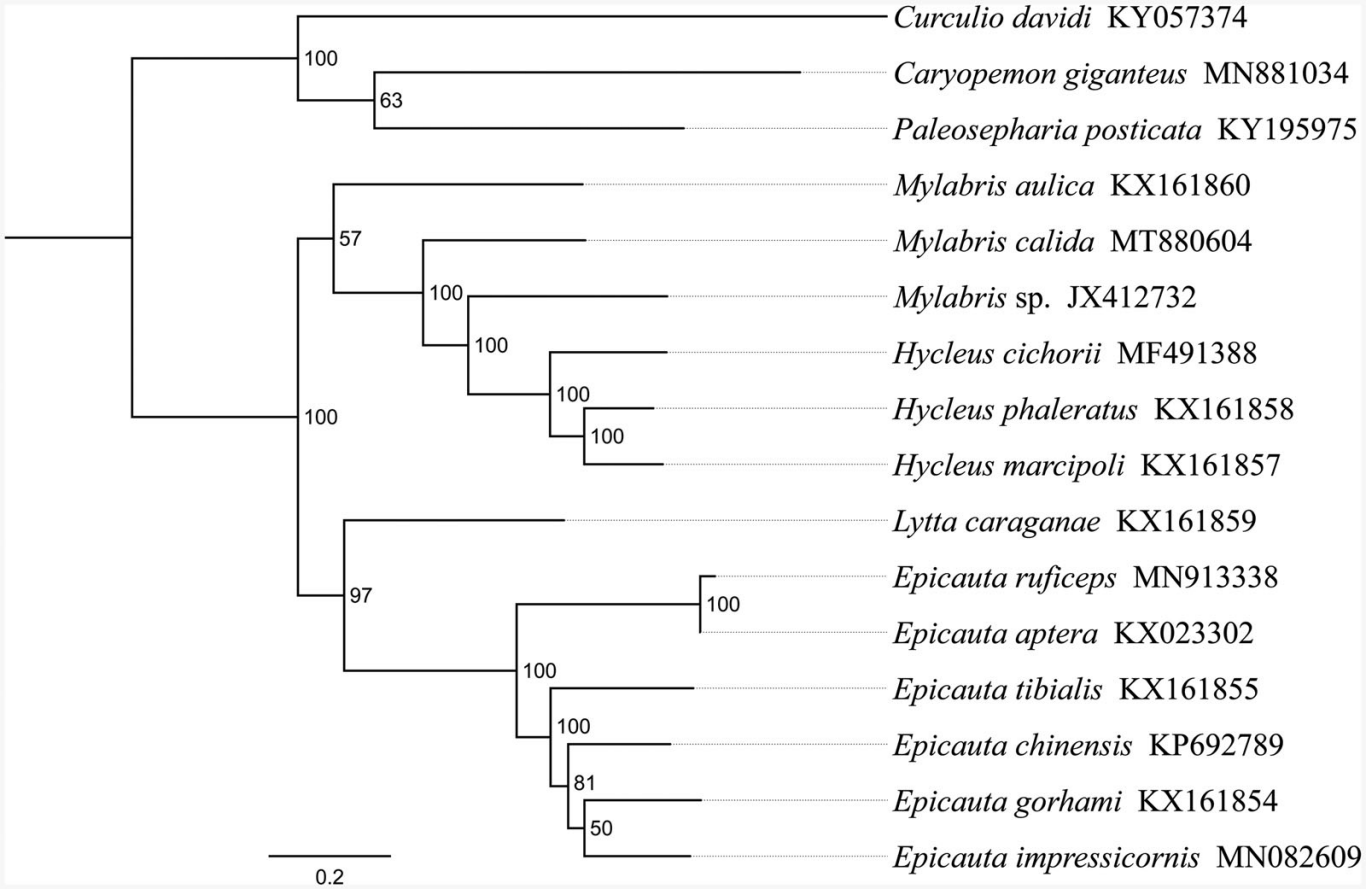
Phylogenetic relationships based on the 13 mitochondrial protein-coding genes sequences inferred from RaxML. Numbers on branches are Bootstrap support values (BS).

## Data Availability

The data that support the findings of this study are openly available in NCBI (National Center for Biotechnology Information) at https://www.ncbi.nlm.nih.gov/, reference number MT880604.
